# Case Report: A case of a pregnancy-associated breast cancer patient with a pathogenic variant in *BRCA1* who underwent staged risk-reducing salpingo-oophorectomy and contralateral risk-reducing mastectomy

**DOI:** 10.3389/fonc.2026.1847327

**Published:** 2026-05-20

**Authors:** Haruko Takuwa, Shoko Sasaki, Naoki Goda, Megumi Takeuchi

**Affiliations:** 1 Department of Breast Surgery, Mitsubishi Kyoto Hospital, Kyoto, Japan; 2 Department of Genetics, Mitsubishi Kyoto Hospital, Kyoto, Japan; 3 Department of Diagnostic Pathology, Mitsubishi Kyoto Hospital, Kyoto, Japan

**Keywords:** BRCA), contralateral risk-reducing mastectomy (CRRM), hereditary breast and ovarian cancer syndrome (HBOC), pregnancy-associated breast cancer, risk-reducing salpingo-oophorectomy (RRSO)

## Abstract

**Background:**

Pregnancy-associated breast cancer in patients with hereditary breast and ovarian cancer syndrome (HBOC) is relatively rare, and reports on treatment outcomes, including those involving risk-reducing surgery, remain limited.

**Case presentation:**

A 38-year-old woman at 8 weeks’ gestation presented with a palpable mass in the left breast. Imaging revealed a tumor in the left breast, and further evaluation led to a diagnosis of luminal B-like, stage IIA left breast cancer. Her family history included a paternal cousin with pregnancy-associated breast cancer at age 37, a maternal grandmother with ovarian cancer at age 68, and a maternal grandaunt with breast cancer. *BRCA1/2* genetic testing identified a pathogenic variant in *BRCA1*. The patient had previously lost her fourth child due to a fetal anomaly (anencephaly) during pregnancy and strongly wished to continue her current pregnancy with her fifth child. Given that neoadjuvant chemotherapy would not affect the surgical approach, that only anthracycline-based therapy can be administered during pregnancy, and that postoperative adjuvant therapy would not be significantly impacted, as well as the potential fetal risks associated with prolonged general anesthesia, contralateral risk-reducing mastectomy (CRRM) was deferred until after delivery. Left total mastectomy with sentinel lymph node biopsy was performed first. As postoperative adjuvant therapy, she received four cycles of doxorubicin and cyclophosphamide during pregnancy, followed by four cycles of docetaxel after delivery, along with tamoxifen and tegafur–gimeracil–oteracil potassium. She underwent staged risk-reducing salpingo-oophorectomy and CRRM after surgery for left breast cancer.

**Conclusions:**

Even in pregnancy-associated breast cancer complicated by HBOC, management should be individualized with careful consideration of safety, and the timing of each intervention should be determined according to the specific clinical circumstances of each patient.

## Introduction

Patients who carry pathogenic variants of the *BRCA1/2* genes have an increased risk of developing breast and ovarian cancers (1, 2). For *BRCA1/2* pathogenic variant carriers, risk-reducing salpingo-oophorectomy (RRSO) is considered an important option because it has been shown to reduce mortality from ovarian cancer, for which effective surveillance methods have not been established (3, 4). Furthermore, it is considered an important option due to its confirmed preventive effect against breast cancer development (5, 6). Based on the results of meta-analyses, RRSO for carriers of pathogenic *BRCA1/2* variants is thought to reduce the risk of developing new breast cancer, at least when performed at age 45 or younger (6). For this reason, the optimal timing for performing RRSO has been considered to be around 35–40 years for *BRCA1* variant carriers and 40–45 years for *BRCA2* variant carriers (7). On the other hand, pregnancy-associated breast cancer is defined as breast cancer that occurs during pregnancy or within 1 year after delivery and is known to carry a significantly higher risk of breast cancer-related mortality compared with nonpregnancy-associated breast cancer (8, 9).

We herein report a case of pregnancy-associated breast cancer with a pathogenic *BRCA1* variant who underwent breast surgery and adjuvant chemotherapy during her pregnancy. All patients diagnosed with hereditary breast and ovarian cancer (HBOC) syndrome in our institution gave their informed consent prior to their inclusion in the study. The ethics committee of our institution approved this study and the use of details from the patients’ medical records (24-17).

## Case presentation

**Figure 1:**
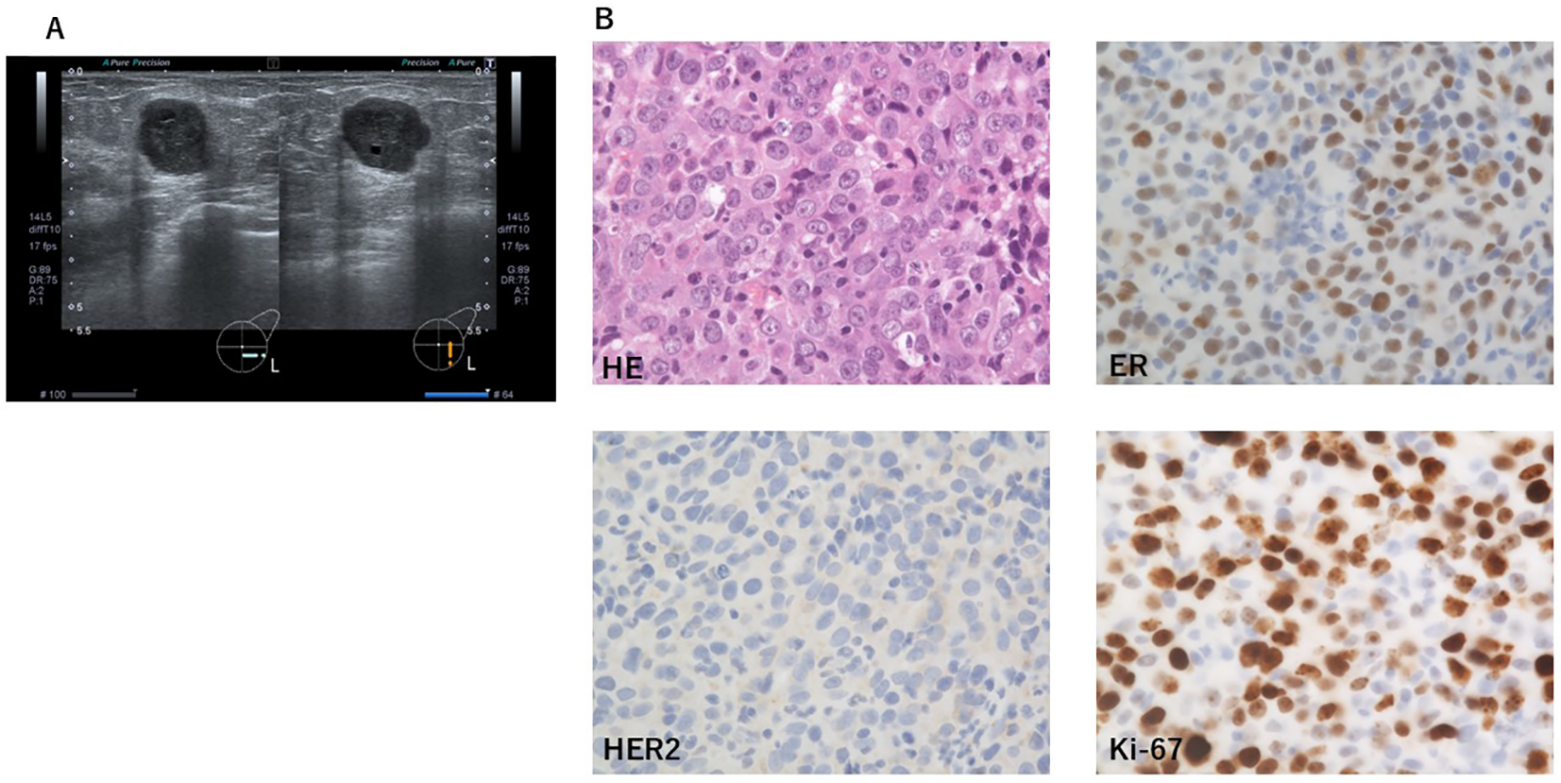
At the diagnosis of the left breast cancer. **(A)** Image of ultrasonography. An irregularly shaped mass was observed in the lower-outer region of the left breast. **(B)** Pathological study revealed the tumor was estrogen receptor (ER) positive (30%), progesterone receptor (PgR) negative (0%), human epidermal growth factor receptor 2 (HER2) negative, with Ki-67 70%, consistent with invasive ductal and medullary breast cancer. HE, hematoxylin–eosin staining; ER, estrogen receptor; PgR, progesterone receptor; HER2, human epidermal growth factor receptor 2.

A 38-year-old woman at her initial visit, currently 8 weeks pregnant with her fifth child, presented with a left breast tumor. A mass was noted in the left breast, and ultrasonography revealed an irregularly shaped mass (**Figure 1A**). Since she was in the first trimester of pregnancy, mammography and magnetic resonance imaging were not performed to avoid potential effects on the fetus. Core needle biopsy confirmed that the tumor was estrogen receptor (ER) positive (30%), progesterone receptor (PgR) negative (0%), human epidermal growth factor receptor 2 (HER2) negative, and Ki-67 70% invasive ductal and medullary breast cancer (**Figure 1B**). A noncontrast computed tomography scan performed after the organogenesis period revealed no evidence of distant metastasis. Radiological assessment classified her as having cT2N0M0, stage IIA disease (UICC-TNM Classification, Ninth Edition).

**Figure 2:**
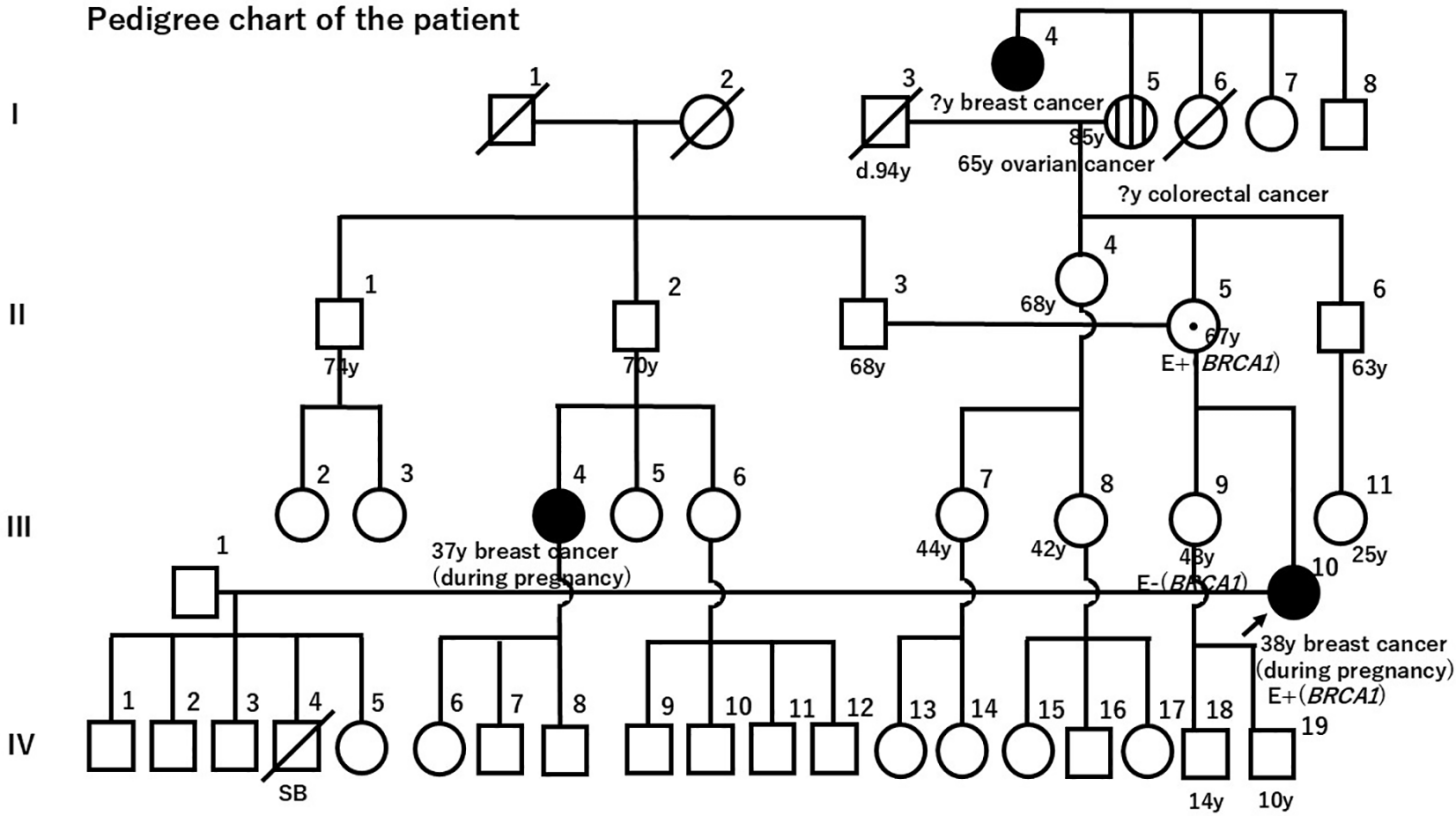
The patient’s family tree. Both the patient and her mother were confirmed to carry the *BRCA1* pathogenic variant.

The patient’s paternal cousin was diagnosed with breast cancer during pregnancy at age 37. The patient’s maternal grandmother developed ovarian cancer at age 68, and the patient’s maternal greataunt had a history of breast cancer (**Figure 2**). BRACAnalysis^®^ confirmed the presence of a *BRCA1* pathogenic variant [c.131_132del(p.Cys44*)]. Additionally, the information was shared with the patient’s sister and mother. Both underwent genetic testing, which revealed that the mother carried the same pathogenic *BRCA1* variant.

**Figure 3:**
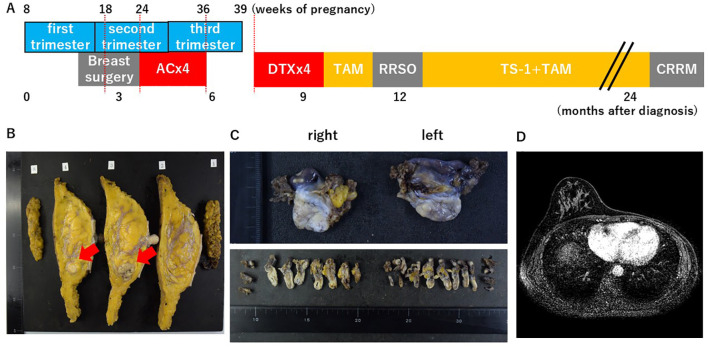
The clinical and pregnancy progress during breast cancer treatment. **(A)** The patient first received a left total mastectomy with sentinel lymph node biopsy. As postoperative adjuvant therapy, she received four cycles of doxorubicin and cyclophosphamide during pregnancy, followed by four cycles of docetaxel after delivery, along with luteinizing hormone-releasing hormone agonist, tamoxifen, and tegafur–gimeracil–oteracil potassium. She was scheduled to undergo staged risk-reducing salpingo-oophorectomy and contralateral risk-reducing mastectomy after surgery for left breast cancer. **(B)** Histological section at the initial left breast mastectomy. **(C)** Histological sections of tissue obtained by risk-reducing salpingo-oophorectomy showed no evidence of malignancy, including serous tubal intraepithelial carcinoma. **(D)** Magnetic resonance images during postoperative surveillance showed no evidence of malignancy in the contralateral breast.

The patient had previously lost her fourth child due to a fetal anomaly (anencephaly) during pregnancy. As she strongly wished to continue her current pregnancy with her fifth child, continuation of the pregnancy was planned. Since neoadjuvant chemotherapy would not affect the choice of surgical procedure and only anthracycline administration is feasible during pregnancy, thereby having a limited impact on postoperative adjuvant therapy, and considering the potential fetal effects of prolonged general anesthesia, CRRM was deferred until after delivery. After thorough consultation with the obstetrician, left mastectomy with sentinel lymph node biopsy was performed upon entering the second trimester, at 18 weeks of pregnancy, 2 months after the breast cancer diagnosis (**Figure 3A**). The final histological analysis diagnosed her as having pT2N0M0, stage IIA disease, histological grade 3, ly0, and v0 (**Figure 3B**). Postoperative adjuvant chemotherapy during pregnancy was planned as four cycles of doxorubicin and cyclophosphamide (AC) between 24 and 36 weeks of pregnancy. Given that the safety of prophylactic pegfilgrastim administration for the fetus in a dose-dense regimen could not be assured, AC was administered every 3 weeks (**Figure 3A**). She delivered her fifth child via vaginal delivery at 39 weeks and 1 day of gestation. It was necessary to initiate postoperative adjuvant chemotherapy after delivery; therefore, the newborn was fed with formula. As the postpartum period was uneventful, postoperative adjuvant therapy with docetaxel (DTX) was resumed 1 month after delivery. After completing four cycles of DTX, RRSO was performed at the patient’s request (**Figure 3C**). No malignant lesions, including serous tubal intraepithelial carcinoma, were identified in the resected tissue. The patient did not meet the criteria of the OlympiA trial (10, 11), and postoperative poly(adenosine diphosphate ribose) polymerase (PARP) inhibitor therapy was therefore deemed not indicated. After completing 1 year of adjuvant chemotherapy with tamoxifen and tegafur–gimeracil–oteracil potassium (TS-1), CRRM was performed. Pathological study revealed no malignant lesions in the right breast tissue. Postoperative endocrine therapy with tamoxifen is being continued. Before CRRM, breast MRI screening was performed at 1 and 2 years postoperatively (**Figure 3D**). No abnormalities in the child’s development were observed at 18 months of age.

The surgical procedures performed in operable breast cancer patients or asymptomatic carriers with pathogenic variants in *BRCA1/2* in our institution are summarized in **Tables 1**, **2**. Case 4 is the patient presented in this case report in **Table 1**. Regardless of *BRCA1/2* variants, 57.1% (12/21) of the patients, after being diagnosed with HBOC, wished to undergo CCRM and RRSO at approximately the same time as their breast cancer surgery. In patients who required intensive systemic treatment for breast cancer, RRSO was often performed after the completion of chemotherapy and other treatments. In **Table 2**, case 3 developed right locally advanced breast cancer at age 33 while being nulligravid and nulliparous. She was diagnosed with HBOC 10 years after surgery; however, due to her desire for childbearing, RRSO was performed at age 44, and CRRM with breast reconstruction was performed at age 45. Patients diagnosed with HBOC at age 60 or older generally did not pursue prophylactic surgery.

**Table 1:** Patient characteristics of individuals with operable breast cancer or asymptomatic carriers of *BRCA1* pathogenic variants.

	Age at genetic testing (y.o.)	Age at diagnosis of breast cancer (y.o.)	Stage at diagnosis	Subtype	Breast surgery (y.o.): right/left	Breast reconstruction: right/left	RRSO (y.o.)
1	25	(−)	(−)	(−)	(−)/(−)	(−)/(−)	(−)
2	37	37	IIIC	ER+/HER2−	CRRM (37)/mastectomy (37)	(−)/(−)	39
3	38	38	I	ER+/HER2−	(−)/breast conserving surgery (38)	(−)/(−)	(−)
4	38	38	IIA	ER+/HER2−	CRRM (40)/mastectomy (38)	(−)/(−)	39
5	45	41	IIA	ER+/HER2−	(−)/mastectomy (41)	(−)/(−)	(−)
	45	45	I	ER−/HER2−	Mastectomy (45)/(−)	(−)/(−)	45
6	47	47	0	ER+	CRRM (47)/nipple-sparing mastectomy (47)	(+)/(+)	47
7	51	51	IIA	ER−/HER2−	(−)/mastectomy (52)	(−)/(−)	(−)
8	53	53	IIA	ER+/HER2−	CRRM(53)/mastectomy(53)	(−)/(−)	54
9	63	(−)	(−)	(−)	(−)/(−)	(−)/(−)	(−)

Case 5, who underwent genetic testing at age 45, was a single individual with a history of metachronous bilateral breast cancer.

RRSO, risk-reducing salpingo-oophorectomy; ER, estrogen receptor; HER2, human epidermal growth factor receptor 2; CRRM, contralateral risk-reducing mastectomy.

**Table 2:** Patient characteristics of individuals with operable breast cancer or asymptomatic carriers of *BRCA2* pathogenic variants.

	Age at genetic testing (y.o.)	Age at diagnosis of breast cancer (y.o.)	Stage at diagnosis	Subtype	Breast surgery (y.o.): right/left	Breast reconstruction: right/left	RRSO (y.o.)
1	34	34	IIIC	ER+/HER2−	CRRM (35)/mastectomy (35)	(−)/(−)	36
2	41	41	IIB	ER+/HER2−	(−)/mastectomy (41)	(−)/(−)	42
3	43	33	IIIC	ER+/HER2−	Mastectomy (33)/CRRM (45)	(−)/(+)	44
4	44	37	IIIB	ER+/HER2−	(−)/breast conserving surgery(37)	(−)/(−)	44
	44	37	I	ER+/HER2−	Breast-conserving surgery (37)/(−)	(−)/(−)	44
5	45	45	I	ER+/HER2−	CRRM (45)/mastectomy (45)	(−)/(−)	46
6	50	50	I	ER+/HER2−	CRRM (50)/mastectomy (50)	(−)/(−)	50
7	51	(−)	(−)	(−)	(−)/(−)	(−)/(−)	(−)
8	51	44	I	ER+/HER2−	Breast-conserving surgery (44)/(−)	(−)/(−)	(−)
	51	51	0	ER+/(−)	CRRM (51)/nipple-sparing mastectomy (51)	(+)/(+)	51
9	57	57	IIB	ER+/HER2−	CRRM (57)/mastectomy (57)	(−)/(−)	58
10	58	58	I	ER+/HER2+	CRRM (58)/mastectomy (58)	(−)/(−)	50^*^
11	63	63	IIB	ER−/HER2−	(−)/mastectomy (63)	(−)/(−)	(−)
12	75	34	I	ER+/HER2−	Mastectomy (34)/(−)	(−)/(−)	(−)
	75	67	I	ER+/HER2+	(−)/breast-conserving surgery (67)	(−)/(−)	(−)

Cases 4, 8, and 12, who underwent genetic testing at ages 45, 55, and 75, respectively, were single individuals with a history of metachronous bilateral breast cancer. Case 10 did not undergo RRSO; instead, she had undergone bilateral salpingo-oophorectomy at age 50 *for ovarian cancer.

RRSO, risk-reducing salpingo-oophorectomy; ER, estrogen receptor; HER2, human epidermal growth factor receptor 2; CRRM, contralateral risk-reducing mastectomy.

## Discussion

In pregnancy-associated breast cancer, diagnostic imaging necessary for breast cancer detection is often restricted, which can lead to significant delays in diagnosis; this may be one of the factors contributing to a poor prognosis (8, 9). It is also necessary to consider the potential harmful effects of radiation therapy on the embryo and fetus, such as the occurrence of malformations due to radiation exposure during the organogenesis period (weeks 3–8 of pregnancy) (12) and a decrease in intelligence quotient during weeks 8–25 of pregnancy, when radiosensitivity is particularly high (13). Additionally, while it is known that patients with homozygous pathogenic variants in the *ATM* gene exhibit extremely high radiosensitivity (7), it is considered that radiation therapy should not be withheld from breast cancer patients who are heterozygous carriers of pathogenic *ATM* variants (14).

We experienced a case of pregnancy-associated breast cancer diagnosed as HBOC. Regarding pregnancy-associated breast cancer with pathogenic *BRCA1/2* variants, there is a report from Israel examining the detection rate of pregnancy-associated breast cancer among *BRCA* pathogenic variant carriers (15); however, its frequency is not high. Although there has been a case report in a Japanese patient describing metastatic disease of pregnancy-associated breast cancer with HBOC (16), reports on the treatment of pregnancy-associated breast cancer diagnosed as HBOC are extremely limited. In this case, we aimed for curative treatment of the newly diagnosed breast cancer and also proceeded with prophylactic surgery for HBOC in accordance with the patient’s wishes.

During pregnancy, even with adequate abdominal shielding, the fetal radiation dose cannot be considered negligible; therefore, radiotherapy is regarded as contraindicated (12, 13). For this patient, given the diagnosis of hereditary breast and ovarian cancer (HBOC), breast-conserving surgery was not planned even if a response to neoadjuvant chemotherapy (NAC) had been achieved. Furthermore, although achieving a pathological complete response after NAC may be associated with a favorable prognosis (17), in this case, it was necessary to ensure both maternal and fetal safety while maintaining the efficacy of cancer treatment. During pregnancy, if the response to chemotherapy is poor and the disease progresses, it is anticipated that making timely adjustments—such as changing regimens or advancing the timing of surgery, as would be done under non-pregnant conditions—would be challenging. Therefore, we considered that prioritizing surgery to remove the tumor from the maternal body, followed by systemic therapy, would offer significant advantages.

Given the high risk of recurrence, adjuvant chemotherapy was planned. Although dose-dense chemotherapy is generally considered effective for young patients with high-risk recurrent breast cancer (18–20), its use was avoided in this case because the effects of pegfilgrastim on pregnancy-associated breast cancer were uncertain. Therefore, adjuvant AC (21–24) followed by DTX (25, 26) was completed with administration every three weeks, ensuring both safety and efficacy. Reviews of taxane administration reported that safety and tolerability for both pregnant women and fetuses were generally maintained (25, 26); however, these findings were based on retrospective studies of only a small number of cases. Since the use of taxanes in pregnancy-associated breast cancer is less common compared to doxorubicin, administration was deferred until after delivery. At the completion of adjuvant chemotherapy, the patient, having no future desire for pregnancy, opted to undergo RRSO. The patient requested an additional 1-year course of TS-1 as adjuvant endocrine therapy (27); therefore, CRRM was performed after the completion of TS-1 administration. Although adjuvant TS-1 is not a standard international regimen, this was chosen according to patient preference and local practice.

Among operable breast cancer patients or asymptomatic carriers of *BRCA1/2* pathogenic variants, the median age of *BRCA1* carriers undergoing CRRM and RRSO was 44 years (range, 37–53) and 45 years (range, 39–54), respectively. For *BRCA2* carriers, the median age was 49 years (range, 35–58) and 47 years (range, 36–58), respectively in our institution (**Tables 1**, **2**). Additionally, no asymptomatic family members opted for prophylactic surgery, partly because such procedures are not covered by health insurance in Japan. On the other hand, patients diagnosed with HBOC at age 60 years or older also tended to avoid prophylactic surgery. Regarding prophylactic surgery, factors such as the desire for future pregnancy and changes in body image mean that it cannot be universally applied to all HBOC patients. However, it is preferable to perform it at a time when its effectiveness is greatest.

High-grade serous ovarian cancers originate from the fallopian tubes; therefore, a comparative trial has been conducted between a group that underwent immediate prophylactic salpingectomy once childbearing was complete and a group that underwent a two-step resection delayed by about 5 years compared with the conventional timing (around 40–45 years for *BRCA1* variants and 45–50 years for *BRCA2* variants). Although there is little difference in cumulative ovarian cancer risk between RRSO and risk-reducing early salpingo-oophorectomy with delayed oophorectomy, clinical trials are ongoing (28). As carriers of *BRCA2* pathogenic variants have a lower risk of developing ovarian cancer compared with *BRCA1* carriers (29), studies are underway to identify patients in whom RRSO may be safely deferred. In this patient, because RRSO could serve as an effective alternative to luteinizing hormone-releasing hormone agonist administration for adjuvant endocrine therapy, and because she had no desire for future childbearing, she opted to undergo RRSO after completion of adjuvant chemotherapy. In our institution, perioperative chemotherapy tailored to the risk of breast cancer recurrence is performed prior to prophylactic surgery for HBOC. In many cases, RRSO is performed either concurrently with breast cancer surgery or within one year after surgery (**Tables 1**, **2**). Because patients have individual perspectives regarding femininity and quality of life, the indication and timing of the procedure are carefully discussed with each patient through shared decision-making.

## Conclusions

While clinical trials to determine the optimal timing for *BRCA1* cases are still underway, it is necessary in clinical practice to propose a timing that is considered appropriate for each case. Even in cases of pregnancy-associated breast cancer with HBOC, proactive treatment with ensured safety is expected to lead to improved survival outcomes.

## Data Availability

The original contributions presented in the study are included in the article, further inquiries can be directed to the corresponding author.
